# NUAK1 links genomic instability and senescence

**DOI:** 10.18632/aging.100153

**Published:** 2010-06-02

**Authors:** David Bernard, Arnaud Augert

**Affiliations:** ^1^ UMR5238, Apoptose, Cancer et Développement, CNRS/Université de Lyon/Centre Léon Bérard, Lyon, France; ^2^ UMR8161, Institut de Biologie de Lille, CNRS/Universités de Lille 1 et 2, Lille, France

**Keywords:** Aneuploidy, senescence, cancer, NUAK1

## NUAK1 and senescence

The AMP-activated protein kinase-related kinase (ARK) family comprises 13 proteins, amongst them NUAK1, that can be classified into five subfamilies: AMP-activated protein kinase (AMPK), salt-induced kinase (SIK), microtubule-affinity-regulating kinase (MARK), brain specific kinase (BRSK), and SNF1-like kinase 1 (NUAK). These proteins regulate biological responses such as metabolism, polarity, cell proliferation or cell death, presumably in a sub-family specific manner [11]. Although the different proteins regulate different responses, their activities are thought to be controlled by the same kinase, LKB1 [2], which phosphorylates a threonine residue in the conserved T-loop of ARK proteins. AMPK proteins are phosphorylated and activated by LKB1 when ATP levels decrease, whereas ARK proteins are phosphorylated and activated independently of intracellular ATP levels [1].

During metabolic stress, ATP levels decrease and LKB1 activates AMPK that, in turn, phosphorylates a subset of proteins. P53 has been identified as one of these proteins, and it is postulated that the phosphorylation and activation of p53 by AMPK leads to cell cycle arrest and senescence [3].

We have recently identified and described the role of NUAK1 in the regulation of replicative senescence. Indeed, the constitutive expression of NUAK1 induces senescence in WI38 normal human fibroblasts whereas its knockdown extends their replicative lifespan. The loss of NUAK1 activation by LKB1 (by using a NUAK1 NUAK1 mutant unresponsive to LKB1 or by inhibiting LKB1 activity in NUAK1 expressing cells) results in a failure of NUAK1 to induce senescence, thus demonstrating the major role of LKB1 in NUAK1-induced senescence. Interestingly, our results support the existence of a p53 independent response, at least in WI38 cells, and emphasize a potential role of aneuploidy in NUAK1-dependent senescence [4].

## Aneuploidy and senescence

Aneuploidy or genomic instability due to various factors have been reported to induce senescence [5, 6, 7]. Interestingly, senescent cells often display elevated aneuploidy, which suggests a putative functional role of aneuploidy in senescence. Nevertheless, it is unclear whether aneuploidy is involved in the establishment of the senescent phenotype and, if prevented, it can impair senescence, at least to some extent. A breakthrough has been achieved with the demonstration that the state of irreversible growth arrest in senescent cells may be due to elevated aneuploidy, putatively through a decrease of LATS1, a kinase involved in mitotic exit [7]. These results suggest that aneuploidy, if not directly involved in the establishment of senescence, can be required for irreversible growth arrest in senescent cells.

Interestingly, aneuploidy was also observed during replicative senescence and during NUAK1-induced premature senescence in our model. More importantly, the replicative lifespan extension due to NUAK1 knockdown correlated with normal ploidy. Altogether, these results suggest that ploidy can be a functional regulator of the senescence program.

We also identified LATS1 as a potential target of NUAK1 and a putative regulator of ploidy in NUAK1-dependent senescence. Altogether, these results suggest that aneuploidy could be part of the endogenous senescence program. Its mis-regulation could therefore induce premature senescence through a process that we chose to term "aneuploidy-induced senescence" (AIS). Our results also suggest that AIS may occur, at least in some settings, without the involvement of the p53 pathway. Interestingly, others have described that the overexpression of Aurora A, a serine threonine kinase tightly associated with the mitotic process, induces senescence in the mammary gland of p53-deficient mice [8]. Hence aneuploidy could be one of the signals triggering senescence and could act, in some settings, independently of p53.

## Aneuploidy-induced senescence as a possible safeguard against tumor formation and development

Oncogene activation is one of the hallmarks of cancer cells and a driving force in tumorigenesis [9]. Oncogene-induced senescence (OIS) was described about a decade ago [10], and a long debate has raged about its relevance. With the development of adequate mouse models of cancer susceptibility and new tools to detect senescence *in vivo*, it has become possible do demonstrate its effectiveness in blocking malignant transformation [11].

Aneuploidy, another classical hallmark of cancer cells, is also believed to be involved in tumorigenesis [12]. As mentioned above, induction of aneuploidy can result in premature senescence in various settings [5, 6, 7]. Together, these observations suggest that AIS, like OIS, could constitute a failsafe mechanism against early tumorigenesis. To validate this hypothesis, it would be interesting to test the presence and the frequency of aneuploidy in benign lesions and to identify the genetic events possibly favoring AIS escape.

The AIS model could resolve the apparent discrepancy about the role of NUAK1 in tumorigenesis. Our recent findings demonstrate an ability of NUAK1 to induce premature senescence in normal human cells whereas others, mainly the team of H. Esumi, have demonstrated a pro tumoral effect of NUAK1 through promoting cell growth and invasion [13, 14, 15]. However, these last conclusions were based on data obtained in cancer cell lines, in particular in colon cancer cell lines known to be highly aneuploid [16]. Thus, NUAK1 may have no additional effect on genomic stability and instead regulate other targets to confer a growth advantage to the cells. NUAK1 might even add more genomic instability, thus conferring additional growth and invasion advantages to these cancer cells.
